# Expression of *Plasmodium falciparum *erythrocyte membrane protein 1 in experimentally infected humans

**DOI:** 10.1186/1475-2875-4-21

**Published:** 2005-04-27

**Authors:** Thomas Lavstsen, Pamela Magistrado, Cornelus C Hermsen, Ali Salanti, Anja TR Jensen, Robert Sauerwein, Lars Hviid, Thor G Theander, Trine Staalsoe

**Affiliations:** 1 Centre for Medical Parasitology at Institute for Medical Microbiology and Immunology, University of Copenhagen, Panum Institute 24-2, Blegdamsvej 3, 2200 Copenhagen N, Denmark; 2 Centre for Medical Parasitology at Department of Infectious Diseases, Copenhagen University Hospital (Rigshospitalet), Copenhagen, Denmark; 3 Radboud University Nijmegen Medical Centre, Nijmegen, The Netherlands

## Abstract

**Background:**

Parasites causing severe malaria in non-immune patients express a restricted subset of variant surface antigens (VSA), which are better recognized by immune sera than VSA expressed during non-severe disease in semi-immune individuals. The most prominent VSA are the *var *gene-encoded *Plasmodium falciparum *erythrocyte membrane protein 1 (PfEMP1) family, which is expressed on the surface of infected erythrocytes where it mediates binding to endothelial receptors. Thus, severe malaria may be caused by parasites expressing PfEMP1 variants that afford parasites optimal sequestration in immunologically naïve individuals and high effective multiplication rates.

**Methods:**

*var *gene transcription was analysed using real time PCR and PfEMP1 expression by western blots as well as immune plasma recognition of parasite cultures established from non-immune volunteers shortly after infection with NF54 sporozoites.

**Results:**

In cultures representing the first generation of parasites after hepatic release, all *var *genes were transcribed, but GroupA *var *genes were transcribed at the lowest levels. In cultures established from second or third generation blood stage parasites of volunteers with high *in vivo *parasite multiplication rates, the *var *gene transcription pattern differed markedly from the transcription pattern of the cultures representing first generation parasites. This indicated that parasites expressing specific *var *genes, mainly belonging to group A and B, had expanded more effectively *in vivo *compared to parasites expressing other *var *genes. The differential expression of PfEMP1 was confirmed at the protein level by immunoblot analysis. In addition, serological typing showed that immune sera more often recognized second and third generation parasites than first generation parasites.

**Conclusion:**

In conclusion, the results presented here support the hypothesis that parasites causing severe malaria express a subset of PfEMP1, which bestows high parasite growth rates in individuals with limited pre-existing immunity.

## Background

*Plasmodium falciparum*-encoded variant surface antigens (VSA) are expressed on the surface of infected erythrocytes (IE) and mediate binding to a range of endothelial cell receptors [1]. Endothelial adhesion contributes to the particular virulence of the *P. falciparum *and most likely has evolved as a mechanism to avoid parasite clearance in the spleen [2-4]. Individuals living in areas of intense parasite transmission develop immunity towards severe malaria early in life [5]. Parasites causing severe malaria in young children with limited pre-existing immunity tend to express a limited, relatively conserved subset of VSA (VSA_SM_) that is more often and better recognized by antibodies from most parasite-exposed individuals than the larger and more diverse VSA_UM _subset expressed by parasites causing uncomplicated malaria [6-8]. It thus appears that expression of VSA_SM _confers a selective advantage in non-immune individuals, perhaps by allowing particularly efficacious endothelial sequestration and consequently high effective growth rates. The best characterized VSA are the *var *gene-encoded *P. falciparum *erythrocyte membrane protein 1 (PfEMP1) family [9-11]. Each haploid parasite genome contains 50–60 *var *genes, of which the 59 *var *genes annotated in the fully sequenced *P. falciparum *clone 3D7 can be divided into three major groups, A, B and C, based on sequence analysis [12,13]. The functional relevance of this grouping is supported by the parallel differences in CD36-binding characteristics of PfEMP1 CIDR1α domains. Thus, GroupA CIDR1α domains do not bind CD36, whereas CIDR1α domains encoded by GroupB and GroupC *var *genes do [14]. The 3D7 PfEMP1 repertoire may well represent the VSA_UM _-VSA_SM _spectrum observed in field isolates, and recent findings point to GroupA as encoding VSA_SM_-type PfEMP1 molecules in patient isolates [13,15-17]. Unfortunately, little is known about *var *gene expression *in vivo*, and studies have been frustrated by the difficulties in detecting and quantifying expression in parasites with unknown *var *gene repertoires. This difficulty has been overcome by taking advantage of the knowledge of the *var *gene repertoire in 3D7 and analyzing *var *gene expression in NF54 parasites (the parental line of 3D7) isolated from non-immune individuals experimentally infected by mosquito challenge. Immediately upon release from the liver, the parasites appeared to transcribe all *var *genes, with GroupA genes being the least transcribed. However, within one or two parasite generations this pattern changed, in particular in those parasites exhibiting the fastest *in vivo *growth rates. Here, only a few genes dominated the *var *transcript population. The data indicate that PfEMP1-determined differences in growth rates shape the expressed PfEMP1 repertoire, and that some PfEMP1 variants confer high effective parasite multiplication rates in non-immune individuals.

## Materials and methods

### Malaria parasites

**Figure 1:**
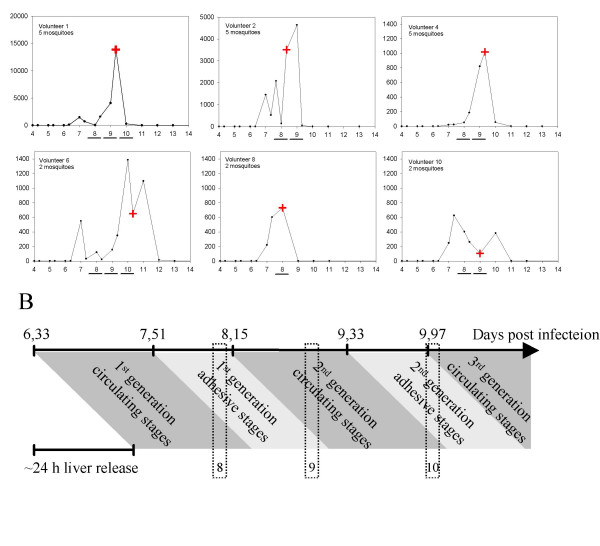
A: Parasite densities in the six volunteers from whom parasite isolates were established. Note that parasitaemia scales are different. Closed circles indicate time points where parasite density was determined by PCR. Time points where blood samples were cultured successfully are underlined. A cross indicates time of chloroquine treatment. B: Representation of parasite generation and stage composition of a *P. falciparum *infection after liver release. Parasites were first detected on day 6,33. Estimates of parasite release from the liver (~24 hours, over all volunteers), duration of circulating stages (1,18 days) and adhesive stages (0,64 days) were taken from reference 24. Time of blood sampling is framed. When sampling blood the early circulating stages are isolated, i.e. blood drawn on day 8 predominantly contains first-generation parasites after liver release.

Parasites were isolated from Dutch volunteers exposed to mosquitoes infected with *P. falciparum *isolate NF54[18] as part of ongoing studies of experimental *P. falciparum *infections. On day 0, ten non-immune volunteers were subjected to two or five infectious bites. Chloroquine treatment was initiated on the first day a thick smear was positive. Parasite cultures were established from 400 microlitres of packed blood cells drawn on days 8, 9, and 10 and parasites were cultured *in vitro *for 27 or 33 days (Figure 1) to obtain sufficient parasite material for DNA, RNA and protein analysis. The parasites were cultured in 0 Rh^+ ^erythrocytes as described [19], with the addition of 2% non-immune human serum to the culture media. Long-term *in vitro *3D7 cultures expressing VSA_UM_-type antigens or selected *in vitro *to express VSA_SM_-type antigens were used as controls[20].

### DNA, RNA, cDNA and quantitative real-time PCR

The development of parasitaemia was monitored by quantitative real-time PCR as described [21]. DNA, RNA and cDNA for *var *gene transcription analysis were prepared from synchronized parasite cultures as described [17]. Quantitative real-time PCR was performed using a Rotorgene thermal cycler system (Corbett Research, Motlake, Australia). Real-time PCR-optimized and gene-specific primers for each of all full-length *var *genes and a pseudogene in the 3D7 isogenic NF54 *P. falciparum *genome were those described in [22], except for *PFI1830c*. Real time primers for this gene were forward 5'ACAACAATTTCGCAAGCAAG 3', reverse 5'TTCCTCTGCCTCCTCTTCAT 3'. Standard curves for the estimation of product-related fluorescent bias and amplification efficiencies were generated for all primer pairs. For 15 primer sets, standard curves were generated both from dilution series of genomic DNA and from cloned gene fragments [17]. As the two approaches led to identical standard curves (not shown), standard curves for the remaining genes were determined from genomic DNA only. The standard curves were linear across a range of seven logs of DNA concentrations (R = 0.9779 to 0.9995) with amplification efficiencies between 90 and 101%. Standard curves were used for primer bias corrections in calculations of absolute transcript levels. The detection limit of the system was ≥ 20 copies. The housekeeping genes *seryl-tRNA synthetase *and *fructose-bisphosphate aldolase *have uniform transcription profiles throughout the parasite life cycle[22], and were used as endogenous controls. Differences in *var *transcript distribution between samples were calculated by the ΔΔ CT method using the endogenous controls for normalization.

### Immunoblot analysis and flow cytometry

Immunoblot analysis was performed as previously described [17]. Flow cytometryand plasma from malaria-exposed children and adults were used to classify the VSA expressed by parasites isolated on days 8, 9 and 10 as previously described[7,20,23]

## Results

### Experimental infections and establishment of parasite culture lines

Successful infections were established in eight of the 10 volunteers. Despite low parasitaemias, a total of 13 parasite culture lines from blood collected from six of the volunteers at three time points after infection were established (Figure 1A). The highest parasite growth rates were observed in volunteers 1 and 2 with peak parasitaemias of 13,832 parasites/ml and 4,637 parasites/ml, respectively, whereas peak parasitaemias in all other volunteers were below 1,400 parasites/ml. As recognized from previous similar human vaccination trials [21], parasitaemias fluctuated in a distinct manner, probably reflecting liver release, sequestration of trophozoite/schizonts and release of new generations of merozoites from schizonts. Parasites obtained on day 8 were predominantly first-generation blood parasites, assuming that parasites were released from the liver between days 6,33 and 7,33[24] (Figure 1B). Similarly, day9 parasites were assumed to represent second-generation parasites, and day10 parasites a mixture of second and third generation parasites.

### Uniform transcription of *var *genes in first-generation asexual parasites

**Figure 2:**
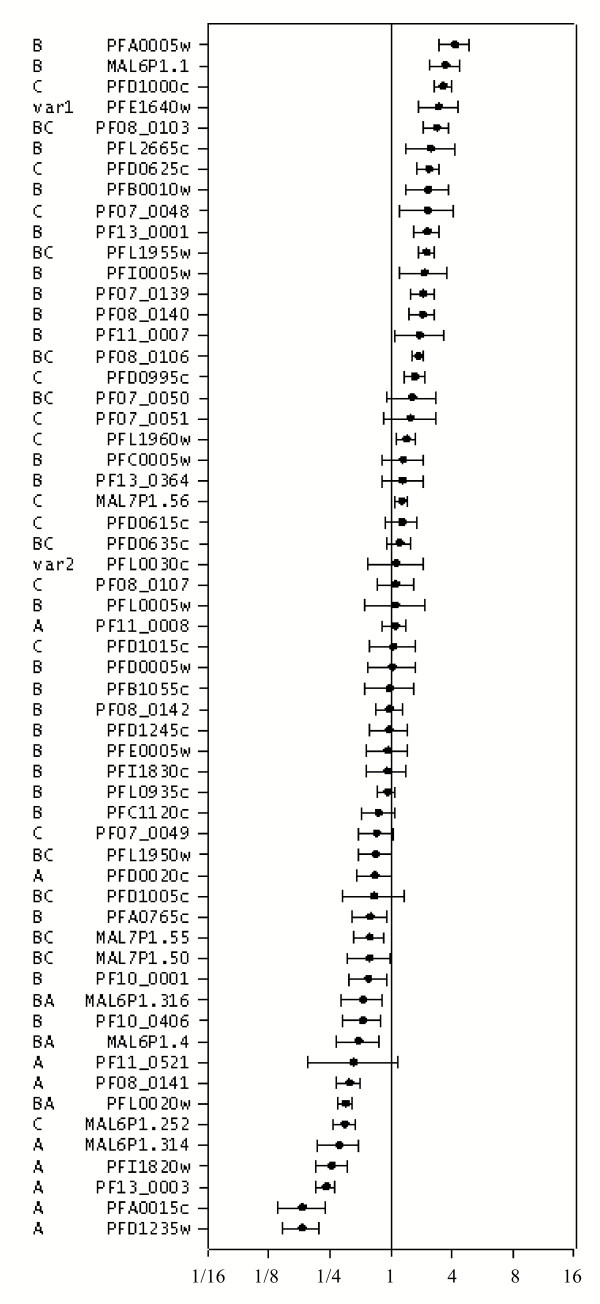
*Var *gene transcription profile of NF54 ring-stage parasite cultures established on day 8 from six volunteers. The mean transcription levels ± 1 SD of primer bias-corrected and normalized values of the six cultures are shown relative to the overall mean *var *transcription level. *Var *gene name and group are indicated.

The pattern of *var *gene transcription was surprisingly consistent in all the six parasite lines obtained on day8 from six of the volunteers (Figure 2). Transcripts of all *var *genes could be detected in ring-stage cultures, and most were transcribed at roughly similar levels. Interestingly, nine of the 10 lowest transcribed genes belonged to *var *subgroup A or B/A, which have previously been associated with severe malaria [13]. In agreement with previous studies [17] all *var *genes were transcribed at markedly lower levels in thetrophozoite/schizont-stage compared to ring-stage parasites (data not shown). The pseudo-gene *PFE1640w *(*var1*) behaved differently and was expressed at similar levels by late stage parasites, comprising approximately 20% of the total number of *var *gene transcripts in trophozoite-stage parasites (data not shown).

### Marked changes in the var gene transcription patterns from the first to the second and third generations of asexual parasites

**Figure 3:**
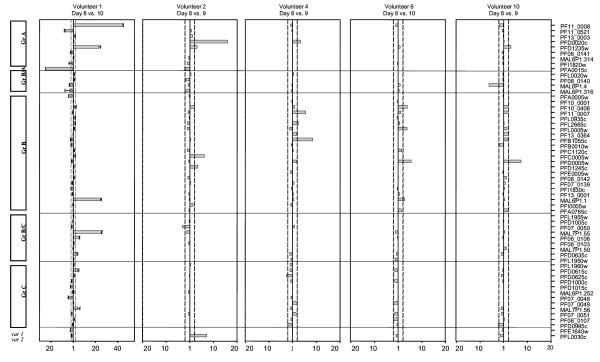
Fold difference in *var *gene transcription between NF54 ring-stage parasites isolated from the same volunteer on different days. Genes are sorted by gene groups as defined in [13]. Note that the fold-change scale for volunteer 1 is different from the other panels. Vertical dashed lines mark an arbitrarily defined two-fold cut off value for biologically significant changes in *var *gene transcription. Experiments with volunteer 1 were repeated three times and results are shown as means ± SD.

The *var *gene transcription patterns of the five isolates obtained on day9 and the two obtained at day10 were different from those of the day8 isolates, in particular in the isolates obtained from volunteers 1 and 2 in whom high parasite growth was observed (Figure 1 and 3). Ring-stage parasites from volunteer 1 showed remarkably large transcriptional changes for five *var *genes. Four (*PF11_0008*, *PFD1235w*, *MAL6P1.1*, *MAL7P1.55*) were transcribed at much higher (>20-fold) and one gene (*PFA0015c*) at markedly lower levels (12-fold) in the day10 isolate compared to the day8 isolate. The estimation of absolute copy numbers (not shown) revealed that *PF11_0008*, *MAL6P1.1 *and *MAL7P1.55 *were the three highest transcribed *var *genes in the day10 isolate, comprising approximately 22%, 40% and 8% of all *var *transcripts, respectively. The abovementioned five genes also showed the most pronounced changes in gene transcription (10–20 fold) when trophozoite stage parasites from days 8 and 10 were compared. The most prominent change among ring-stage parasites from volunteer 2 was the 15-fold increased transcription of *PFD0020c*, which was the highest (~8%) transcribed *var *gene in the day 9 isolate (Figure 3). Only minor changes in *var *gene transcription patterns between days 8 and 9/10 were observed in the parasites isolated from volunteer 4, 6 and 10.

Overall, none of the *var *genes had a consistently altered transcript proportion in all volunteers. However, transcription of seven genes (*PFD0005w*, *PFD0020c*, *PFD1235w*, *MAL6P1.1*, *PF10_0406*, *PF11_0007 *and *PFL0005w*) increased in more than one volunteer, whereas transcription of three genes (*PFA0015c*, *MAL6P1.4 *and *PFE1640w*) decreased in more than one volunteer (Figure 3). In general, analysis of ring-stage and trophozoite/schizont-stage parasites yielded similar results.

### Expected changes in PfEMP1 expression between first, second and third generation asexual parasites were confirmed by western blot analysis

**Figure 4:**
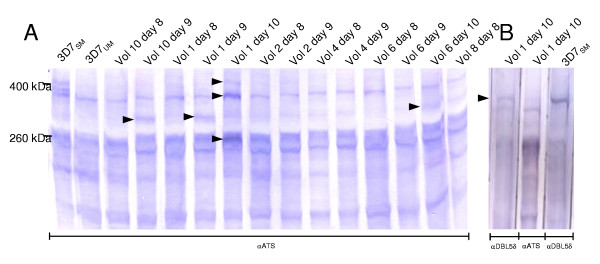
PfEMP1 expression in trophozoite-stage cultures of unselected 3D7 (3D7_UM_), 3D7 selected for expression of VSA_SM_-type IE surface antigens (3D7_SM_) and of NF54 established from six volunteers on different days after infection. A: Western blot using antibodies (αATS) targeting the acidic terminal segment (ATS), which is conserved between most PfEMP1 types. Black arrows indicate changes in protein expression between isolates of the same volunteer. B: Western blot identifying the αATS-detected 400 kDa band in the day 10 culture of volunteer 1 as PFD1235w/VAR4 using an αDBL5δ antibody. VAR4 is expressed on the surface of the 3D7_SM _line selected for high immune serum recognition [17].

To investigate PfEMP1 translation in the 13 isolates, western blot analysis were performed on protein extractions of trophozoite/schizont-stage cultures using rabbit and murine antisera raised against the conserved acidic terminal segment (ATS) and against DBL5δ of *PFD1235w*, respectively (Figure 4). The analysis of parasites from volunteer 1 revealed differential expression from day8 to day10 (Figure 4A). Thus, a high molecular weight band of around 400 kDa was seen only in the day 10 culture when using the ATS-specific antibody (Figure 4A). This band size corresponds to the expected size of PFD1235w, which also exhibited a large increase in transcription from day8 to day10 among parasites from this donor, and its identity was confirmed using the PFD1235w DBL5δ antibody (Figure 4B). PFD1235w could not be detected in any of the other cultures, although small increases in transcription were found in several of the cultures (not shown). Two bands of 350 kDa and 260 kDa were particularly intense in the day10 culture of volunteer 1 (Figure 4A). This finding correlates with the expected sizes and elevated transcription of *PF11_0008 *(345 kDa), *MAL6P1.1 *and *MAL7P1.55 *(both ~258 kDa). Transient bands at around 230 kDa and 310 kDa appeared to emerge on day 9 and disappear on day 10 in the cultures of volunteer 1. No obvious identity could be assigned to these proteins. In the remaining cultures, differential PfEMP1 expression detected by the ATS antibody was found in volunteers 6 and 10. In both cases a band of 310 kDa was observed in day10 and 9 parasites, respectively. The best candidate gene for this band is *PFD0005c*, which is predicted to have the observed size and was found to be more highly transcribed in the these cultures. All day 8 cultures were very similar and all appeared to dominantly express PfEMP1s around 260 kDa, which corresponds to the expected sizes of the highest transcribed genes in these cultures (figure 2).

### Second and third generation parasites express VSA that are recognized more frequently by immune plasma than first generation parasites

**Figure 5:**
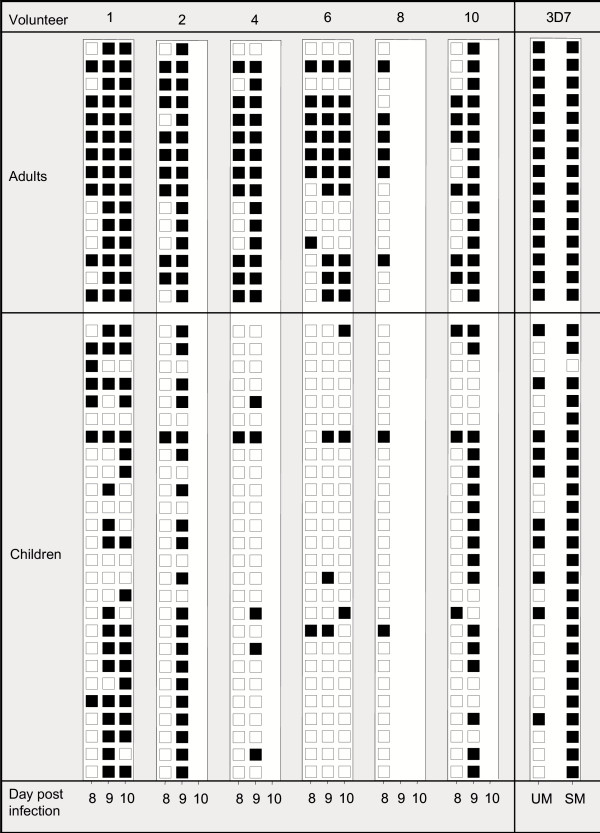
Plasma recognition profiles of trophozoite-stage cultures of NF54 established from six volunteers on different days after infection. Profiles of unselected 3D7 (UM) and 3D7 selected *in vitro *for expression of VSA_SM_-type IE surface antigens (SM) are shown for comparison. Recognition was measured by flow cytometry using IgG from Ghanaian adults and children (see Materials and Methods). Filled boxes indicate mean FITC-fluorescenceindex (MFI)abovea cut-off defined by the mean + 2 standard deviations of 8 Danish control plasma.

The VSA phenotype of parasites can be classified relative to each other and in the spectrum between VSA_SM _and VSA_UM_, depending on the proportion of individuals from an endemic area that possess antibodies to the expressed VSA [7,20]. To establish their VSA phenotype, the serological recognition of the isolated NF54 parasites was tested using a panel of plasma obtained from children and adults living in Coastal Ghana (Figure 5). All day 8 parasite isolates, representing the first generation of asexual blood-stage parasites, expressed VSA that were recognized by IgG in plasma samples from only a minority of the children and from about half of the adults (Figure 5). These parasites were also less well recognized than the standard 3D7 VSA_UM _line, which dominantly expresses PfEMP1 encoded by GroupC *var *genes. The seven lines isolated on days 9 or 10 all expressed VSA that were more frequently recognized than the corresponding day8 line (P = 0.01, Wilcoxon signed-ranked test). This trend was particularly clear for the day9/10 lines from volunteers 1, 2 and 10, which were recognized by all of the plasma samples from adults and from the majority of the children, similarly to the recognition of 3D7 selected for expression of VSA_SM_-type IE surface antigens (Figure 5). However, quantitatively the serum recognition of all isolates was lower than that of the 3D7_SM _line (data not shown). The 3D7 VSA_SM _line has been selected for VSA_SM _expression using IgG from semi-immune children and dominantly expresses the product of *PFD1235w *(VAR4) on the surface on infected erythrocytes. Interestingly, parasites from volunteers 1, 2 and 10 had an increased transcription of a particular *var *gene (*PFD1235w*; *var4*) (Figure 2) as does 3D7 in response to selection for VSA_SM _expression[17].

## Discussion

The inter- and intra-clonal variability of the *var *genes have frustrated attempts to investigate the roles of the encoded PfEMP1 proteins in pathogenesis and protection. The most common strategy has been to quantify *var *transcription by counting the frequency of unique sequence tags, amplified by degenerate primers targeting semi-conserved blocks of DBL domains. This has been used to study phenotypically distinct laboratory lines [10,25-29]and parasite strains isolated from patients with defined clinical outcomes [16,30-32] The only previous study of *var *gene transcription in experimentally infected humans also applied this strategy [33]. However, it requires the sequencing of a large number of clones for statistical significance and is inherently susceptible to primer bias. Together, this makes data interpretation difficult. To overcome these difficulties, sensitive and gene-specific tools were used to analyse in detail the pattern and dynamics of *var *gene expression in non-immune volunteers infected with a parasite with a known *var *gene repertoire. The necessity for *in vitro *expansion of parasites from blood samples with submicroscopic parasitaemias makes this approach susceptible to two separate types of bias. Firstly, cultures were established from a relatively small number of parasites and the transcription profiles of these parasites may not represent the profile of the entire *in vivo *population. However, in most cultures the founder population was between 100 and 4,000 parasites, and only two cultures, which did not exhibit biased transcription patterns (day 8 culture of volunteers 1 and 4) were established from less than 100 parasites. Secondly, *P. falciparum *has been reported to switch *var *gene expression at variable rates *in vitro *[34,35]. Hence, the *var *gene expression in the cultures at the time of transcription analysis may not reflect the expression profile *in vivo *at the time of blood collection. The transcription profiles of the cultures isolated on day 8 were similar and different to the profiles of the parasites isolated on days 9 and 10. This, and the fact that the differential *var *gene transcription, translation and serological recognition of PfEMP1 correlated with parasite growth, however, indicates that *in vitro *switching did not invalidate the analyses.

No *var *gene was dominantly transcribed in the day 8 cultures. Instead, a relatively large group of genes belonging to *var *groups B and C were expressed at almost similar levels and interestingly, nine of the 10 lowest transcribed genes belonged to group A or B/A. These data imply that the PfEMP1 expression pattern at the beginning of the infection is broad and anticipatory. In addition, the consistent increase in recognition by immune sera with time of infection and the apparent association between high growth rates and differential expression of a few group A and B genes from first to second and third generation imply that it is the host environment that modulates the PfEMP1 expression. This is a plausible scenario because of the large and immediate differences in survival fitness that are likely to be imposed on the asexual parasites by the physiology and pre-existing immunity of the host. While most – or all – of the PfEMP1 variants that can be expressed by a given parasite will be exposed to the immune system according to this model, the majority of the variants are likely to be present too briefly to induce a significant immune response. Crucially, survival fitness differences depend on which PfEMP1 variants are being expressed and necessitate a parasite response much faster than which can be achieved by switching to advantageous *var *genes. Although differences in switching rates can contribute to the pattern of *var *gene expression [35] and may well be responsible for the differences in transcription observed on day 8 (Figure 2), differences in survival rates may be far more important in focusing and ordering PfEMP1 expression *in vivo*. In non-immune individuals this process would be expected to focus expression on the restricted and relatively conserved subset of VSA (VSA_SM_) associated with severe disease in patients with little pre-existing immunity [13,17] It has previously been documented that *in vitro *selection of 3D7 for acquisition of the VSA_SM _phenotype is associated with expression of a subset of *var *genes [[15]. Strikingly, four of the five marked differentially transcribed genes (*PF11_0008*, *PFD1235w*, *MAL7P1.55 and PFA0015c*) in the volunteer 1 cultures, were among the few genes differentially transcribed genes upon selection for the 3D7_SM _phenotype, in which there was selection for expression of *PF11_0008*, *PFD1235w *and *MAL7P1.55 *and against expression of *PFA0015*.

In the study of Peters et al [33] the clone frequency strategy was used to investigate *var *transcription profiles of parasites isolated from two volunteers on day 12 and 13 after infection with 3D7 by eight or nine infectious mosquito bites. One transcript, PF11_0007, belonging to *var *group B, comprised half of the 39 and 41 sequences cloned from the two volunteers respectively. In total 10 and nine different *var *tags were found and only one belonged to *var *group A. The parasites were predicted to be 3rd or 4th generation and the parasitaemias in the two volunteers were 18,000 and 212,000 parasites/ml. Thus, these profiles are best compared with that of the day 10 isolate of volunteer 1 presented in this study. Taking into account the potential ambiguities in the interpretation of the results presented by Peters et al, we believe that the two data sets could reflect similar dynamics in the human host.

## Conclusion

In conclusion, the data – in combination with earlier findings – suggest that PfEMP1 expression is determined and ordered mainly by host physiology and immunity, and that this will cause infections in non-immune individuals to be dominated by VSA_SM_-type variants such as those encoded by Group A and B *var *genes.

## Abbreviations

PfEMP1 *Plasmodium falciparum *erythrocyte membrane protein 1

VSA_UM _variant surface antigens associated with uncomplicated malaria

VSA_SM _variant surface antigens associated with severe malaria

## Authors' contributions

TL carried out the transcription analysis, took part in the parasite sampling and culturing and prepared the manuscript. PM performed the western blot analysis. CCH ran the human vaccination trials. TS managed the sample collection, culturing of parasites as well as performed and the flow cytometry analysis. TL, CCH, TGT and TS conceived the design of the study. All authors helped to draft and approved the final manuscript.
